
JOURNAL INFORMATION
==============================
NLM Title Abbreviation: ScientificWorldJournal
Journal ID: ScientificWorldJournal
NLM Journal ID: 101131163
Journal ID: TSWJ

The Scientific World Journal

ISSN: 2356-6140
EISSN: 1537-744X
Publisher: Wiley

ARTICLE INFORMATION
==============================
PMCID: PMC6009384
PMID: 29973794
DOI: 10.1100/tsw.2002.20
Article ID: 592189
Article version: 1
Subjects: Short Report

Purification of Gst Fusion Proteins, On-Column Cleavage, and Sample Clean Up

Sigrell Jill A.
Heijbel Anna *
Amersham Pharmacia Biotech AB, SE-751 84 Uppsala, Sweden
*Anna Heijbel: anna.heijbel@eu.apbiotech.com
Publication date: 2002
Electronic publication date: 2002 Mar 27
Volume: 2
First page: 39
Last page: 40
Copyright: Copyright © 2002 Jill A. Sigrell and Anna Heijbel.
Copyright year: 2002
License: This is an open access article distributed under the Creative Commons Attribution License, which permits unrestricted use, distribution, and reproduction in any medium, provided the original work is properly cited.
License URL: https://creativecommons.org/licenses/by/3.0/

==============================
Advances in Gene Technology: The Genome and Beyond –
Structural Biology for Medicine (Proceedings of the 2002 Miami
*Nature Biotechnology* Winter Symposium)
*TheScientificWorld* 2002, 2(S2), 39–40
ISSN 1532-2246; DOI 10.1100/tsw.2002.20

### PURIFICATION OF GST FUSION PROTEINS, ON-COLUMN CLEAVAGE, AND SAMPLE CLEAN UP

Jill A. Sigrell and Anna Heijbel
Amersham Pharmacia Biotech AB, SE-751 84 Uppsala, Sweden
anna.heijbel@eu.apbiotech.com

**INTRODUCTION.** Expression of recombinant proteins fused to the affinity tag Glutathione S-transferase (GST) allows fast and easy protein purification by chromatography on Glutathione Sepharose™[1,2]. This work, we purify six different human proteins all produced as fusions to GST and expressed in *E. coli*.

**METHOD AND RESULTS.** Four of the proteins are part of a structural genomics project, where the aim is to determine their 3-dimensional structures. Firstly, the proteins were produced on a small scale. Using a preprogrammed template on ÄKTA™*prime*, the purification of these four proteins from cell extracts was quickly performed, making it possible to easily evaluate each protein's expression, purity, and stability. This information will, for each protein, enable a scale-up strategy for expression and purification that is required to produce enough homogenous material for structural studies.

The other two proteins, were expressed using two different pGEX vectors which introduce protease sites between the GST affinity tag and the target protein. With this approach pure, free target protein can be obtained in a straightforward way. The vector expressing hippocalcin, a member of the neurone-specific, calcium-binding protein family, has a PreScission™ protease site. By on-column cleavage using this GST tagged protease, 10 mg of pure hippocalcin was obtained from 1 g of wet cells.

When expressing the SH2 domain from a phosphatase, a thrombin site was incorporated between the GST tag and the SH2 domain. Thrombin (a serine protease that can bind the synthetic inhibitor p-aminobenzamidine) could be completely removed after on-column cleavage by placing a HiTrap™ Benzamidine FF (high sub) column in series after the GSTrap™ FF column. The removal of thrombin was verified with an activity assay using the substrate S-2238. From 1 g of wet cells, 5 mg of pure SH2 domain was obtained.

**SUMMARY.** All recombinant proteins were purified using prepacked GSTrap FF columns. Removal of the GST tag using an appropriate protease was easily performed on column and additional affinity columns allowed cleaved target proteins to be eluted without contaminating proteases.

**ACKNOWLEDGEMENT.** Dr. Kristina Bäckbro, Uppsala University, Uppsala, Sweden for GST-hippocalcin, Pharmacia Corporation, Stockholm, Sweden for SH2-GST and Martin Hammarström, Royal Institute of Technology, Stockholm, Sweden for GST-fused ORFs.

### REFERENCES

1. Amersham Pharmacia Biotech (2001) GST Gene Fusion System Handbook.
2. Amersham Pharmacia Biotech (2001) The Recombinat Protein Handbook.
